# The Royal Commission Report

**Published:** 1912-08-10

**Authors:** 


### THE DEFECTIVE AND DEPENDENT.*

### XI.—The Royal Commission Report.

### THE LEGISLATIVE PROPOSALS FOR THE CARE OF THE FEEBLE-MINDED.

In September 1904 a Royal Commission was appointed "to consider the existing methods of dealing with idiots and epileptics, and with imbecile, feeble-minded, or defective persons not certified under the Lunacy Laws," and to report what amendments in the law were required to secure their due care, training, and control.

This Commission examined no less than 248 witnesses, visited institutions at home and abroad for those classes of the population, making special medical investigations in certain selected districts in Great Britain and Ireland, and on July 31, 1908, published the result of their labours in eight voluminous Blue-books. In effect it was estimated that in England and Wales alone the number of mentally-defective persons, including certified lunatics, idiots, and imbeciles, was 271,607, and that of these 149,628 were feeble-minded persons not considered certifiable under the Lunacy Laws. For at least 66,509 of the latter provision was urgently needed. In four typical urban areas investigated 12.7 per cent. of those in Poor-Law institutions were mentally defective, and the percentage was 18.75 in those of four rural districts. Instances of feeble-minded women, with illegitimate and sometimes feeble-minded progeny, were found not infrequently in the wards of workhouses, and yet there was no legal means of detaining them, as the medical officers did not consider them certifiable under the Lunacy Laws. Amongst the juvenile population an average of at least 5.9 per thousand on the rolls of elementary schools were considered to be mentally defective, giving a total of 35,662 such children in the elementary schools of England and Wales. Of this number only about one-fourth were then provided for in special schools, which were very unevenly distributed, restricted mainly to large towns, and as the Education (Defective and Epileptic Children) Act, 1899, was adoptive and not compulsory many districts were entirely destitute of special school accommodation. Moreover, at the age of sixteen the powers of the education authorities over such children ceased, and consequently at the most perilous period of life the young people who had passed through the special schools were turned adrift on the world. In prisons, rescue homes, inebriate and other institutions, a considerable proportion of mentally-defective inmates were to be found. The Commissioners well sum up the results of the evidence they gathered in one of the opening paragraphs of their Report as follows:—"Of the gravity of the present state of things there is no doubt. The mass of facts that we have collected, the statements of our witnesses, and our own personal visits and investigations compel the conclusion that there are numbers of mentally-defective persons whose training is neglected, over whom no sufficient control is exercised, and whose wayward and irresponsible lives are productive of crime and misery and continuous wasteful expenditure."

#### Recommendations.

The main principles upon which the recommendations of the Commission are based may be shortly stated as follows:—

1. That persons who cannot take a part in the struggle of life, owing to mental defect however designated, should be afforded by the State such special protection as may be suited to their needs.

2. That the mental condition of such persons, and not their poverty or crime, is the real ground for help from the State.

3. That it is necessary to ascertain who are mentally defective and where they are, so as to bring them into relation with the local authority.

4. That the protection of the mentally-defective person should be continued so long as it is necessary for his good.

5. That to supervise local administration a central authority is indispensable.

6. That in regard to the protection of property all mentally-defective persons should have like privileges, and that the Chancery Division of the High Court, acting in concert with the supervising authority, should be charged with this duty.

The recommendations consequently suggest the formation of a single central authority for the supervision of all classes of mentally-defective persons, including lunatics, working under a single Act, and having jurisdiction over the following:—(1) Persons of unsound mind; (2) persons mentally infirm from decay of their faculties; (3) idiots; (4) imbeciles; (5) feeble-minded; (6) moral imbeciles; (7) epileptics; (8) inebriates; (9) deaf and dumb or blind (those of the last three categories being also mentally defective). It is suggested that the central supervising authority (called the Board of Control) should be practically an extension of the present Lunacy Commission, and that, in addition, Assistant District Commissioners should be appointed in at least eight divisions of England and Wales to assist in supervision and visitation. County and County Borough Councils are to appoint a statutory committee for the care of mental defectives, which is to ascertain the number of mentally-defective persons for whom the council is liable to provide, and in the case of educable feeble-minded children this committee is to be the supervising authority of the special schools, which are to be certified by the Board of Control instead of, as at present, by the Board of Education.

No action was taken on the above Report until the present session of Parliament, but the "Mental Deficiency Bill" and the "Feeble-Minded Control Bill" (promoted by the National Association for the Feeble-Minded) have severally passed second reading, so that the prospect of special legislation more or less on the lines of the Royal Commission Report seems at length in sight.

* Previous articles appeared on Nov. 4, 18, Dec. 2, Jan. 6, Feb. 10, March 2, 23, May 4, June 15, July 13.

